# Predicting the prognosis in patients with sepsis by a pyroptosis-related gene signature

**DOI:** 10.3389/fimmu.2022.1110602

**Published:** 2022-12-21

**Authors:** Shuang Liang, Manyu Xing, Xiang Chen, Jingyi Peng, Zongbin Song, Wangyuan Zou

**Affiliations:** ^1^ Department of Anesthesiology, Xiangya Hospital, Central South University, Changsha, Hunan, China; ^2^ National Clinical Research Center for Geriatric Disorders, Xiangya Hospital, Central South University, Changsha, Hunan, China

**Keywords:** sepsis, pyroptosis, gene, prediction, prognosis, diagnosis

## Abstract

**Background:**

Sepsis remains a life-threatening disease with a high mortality rate that causes millions of deaths worldwide every year. Many studies have suggested that pyroptosis plays an important role in the development and progression of sepsis. However, the potential prognostic and diagnostic value of pyroptosis-related genes in sepsis remains unknown.

**Methods:**

The GSE65682 and GSE95233 datasets were obtained from Gene Expression Omnibus (GEO) database and pyroptosis-related genes were obtained from previous literature and Molecular Signature Database. Univariate cox analysis and least absolute shrinkage and selection operator (LASSO) cox regression analysis were used to select prognostic differentially expressed pyroptosis-related genes and constructed a prognostic risk score. Functional analysis and immune infiltration analysis were used to investigate the biological characteristics and immune cell enrichment in sepsis patients who were classified as low- or high-risk based on their risk score. Then the correlation between pyroptosis-related genes and immune cells was analyzed and the diagnostic value of the selected genes was assessed using the receiver operating characteristic curve.

**Results:**

A total of 16 pyroptosis-related differentially expressed genes were identified between sepsis patients and healthy individuals. A six-gene-based (*GZMB, CHMP7, NLRP1, MYD88, ELANE*, and *AIM2*) prognostic risk score was developed. Based on the risk score, sepsis patients were divided into low- and high-risk groups, and patients in the low-risk group had a better prognosis. Functional enrichment analysis found that NOD-like receptor signaling pathway, hematopoietic cell lineage, and other immune-related pathways were enriched. Immune infiltration analysis showed that some innate and adaptive immune cells were significantly different between low- and high-risk groups, and correlation analysis revealed that all six genes were significantly correlated with neutrophils. Four out of six genes (*GZMB, CHMP7, NLRP1*, and *AIM2)* also have potential diagnostic value in sepsis diagnosis.

**Conclusion:**

We developed and validated a novel prognostic predictive risk score for sepsis based on six pyroptosis-related genes. Four out of the six genes also have potential diagnostic value in sepsis diagnosis.

## Introduction

Sepsis is a dysregulation of the host’s response to infection, which is frequently accompanied by life-threatening organ dysfunction (1, 2). Despite advances in our understanding of sepsis, supportive therapies including early fluid resuscitation, the use of antibiotics, and the provision of supportive care for organ function have remained the standard of care, and the mortality rate of sepsis is still high, reaching about 26% (3, 4). Early diagnosis and intervention those sepsis patients who are associated with increased mortality risk are critical for a better prognosis (5). Therefore, it is important to explore diagnostic and prognostic signatures in sepsis patients.

Pyroptosis is a novel form of proinflammatory and programmed cell death, which also participates in the response of the innate immune system (6). With the clarification of the mechanism of pyroptosis, it is now considered that pyroptosis is mediated by the activation of the gasdermin-D (GSDMD) protein *via* the active caspase-1 (canonical pathway) or the active caspase-4/5/11 (non-canonical pathway), which causes cell swelling, rupture, and the release of inflammatory cytokines such as Interleukin 18 (IL-18) and Interleukin 1 β (IL-1β) (7–9). Previous studies demonstrated that pyroptosis may play an important role in the development of sepsis and sepsis-related organ dysfunction including acute kidney injury (10), acute lung injury (11), cardiac dysfunction (12), and disseminated intravascular coagulation (13). Therefore, pyroptosis-related genes were recognized as promising therapeutic targets of sepsis (14), and treatment by using non-specific or specific caspase inhibitors has shown therapeutic effects in experimental studies (15, 16). However, few studies focused on the value of the pyroptosis-related gene in predicting the prognosis and diagnosis of sepsis, and the prognostic and diagnostic value of pyroptosis-related genes have not been fully investigated.

In this study, we identified molecular subtypes of sepsis based on pyroptosis-related genes, developed and validated a novel pyroptosis-related prognostic risk score for sepsis patients, and investigated the correlation between pyroptosis-related genes and the immune cells. Based on the prognostic risk score and clinical characteristics of sepsis patients, a nomogram was created, and the diagnostic value of pyroptosis-related genes was also assessed. Our findings may provide new insight into the role of pyroptosis in the prognosis and diagnosis of sepsis.

## Materials and methods

### Microarray data and data process

Two peripheral-blood gene expression datasets (GSE65682 and GSE95233) and their corresponding clinical data were obtained from Gene Expression Omnibus (GEO) database (https://www.ncbi.nlm.nih.gov/gds/). The GSE65682 dataset and GSE95233 dataset were based on GPL13667 and GPL570 platforms, respectively. The GSE65682 dataset comprised 479 sepsis patients with complete data for survival status within 28 days and 42 healthy controls. Sepsis patients in GSE65682 were separated into the discovery cohort (n = 263) and validation cohort (n = 216) for its original investigation and we used these two cohorts for survival analysis in our study. The GSE95233 dataset comprised 51 sepsis patients and 22 healthy controls. Only the gene expression data of the blood sample collected at admission to the intensive care unit was used for analysis in the present study.

The gene probe was transformed into gene symbol by using the corresponding annotation profile in each dataset. We used ‘limma’ package in R software to quartile normalized for all gene expression values and generate normally distributed expression values. For multiple same probes, the finial gene expression value was determined by calculating the average expression value.

### Screening pyroptosis-related differentially expressed genes and consensus clustering analysis

A total of 60 pyroptosis-related genes were identified from gene set enrichment analysis (GSEA) website (http://www.gsea-msigdb.org/gsea/index.jsp) and previous literature (17–21) (**Supplementary Table 1**). Differentially expressed genes (DEGs) between sepsis and healthy samples in GSE65682 were screened using the ‘limma’ package, with log_2_|fold change| > 0.5 and adjust *P* value< 0.05 set as cut-off criteria to screen DEGs (22, 23). Pyroptosis-related DEGs were identified by intersecting the DEGs with the pyroptosis-related genes. A protein-protein interaction (PPI) network analysis was conducted by using the STRING website (https://cn.string-db.org/) to further explore the interaction between these pyroptosis-related genes. Based on the expression value of pyroptosis-related DEGs, we used “ConsensusClusterPlus” package to identify the molecular subtype of sepsis. The pam algorithm with euclidean distance was used, and the samples were iterated 1000 times. The k value was increased from 2 to 6 to identify the optimal clusters.

### Identification of survival-related pyroptosis-related genes and construction of a prediction model for prognosis

Univariate Cox regression analysis was performed in the discovery cohort to evaluate the prognostic value of each pyroptosis-related DEGs. To avoid omissions, we set a *P*-value lower than 0.2 as a significant cut-off value (24). Pyroptosis-related DEGs with a significant correlation to survival status were selected as candidate genes for further investigation. Then, we used LASSO-Cox regression *via* ‘glmnet’ packages to screen the candidate genes and construct the prediction model. The penalty coefficient λ was determined by using the minimal criteria and candidate genes with a regression coefficient unequal to zero were included in the final model. The risk score of each case was calculated according to the following formula: $\text{Risk score }=\sum_{i}^{k}\text{Coefficient i }\times \text{ Gene Expression value of i}\;$ . After that, sepsis patients were divided into low- and high-risk groups based on the median risk score, and the survival time between the two risk groups was compared using Kaplan-Meier analysis. Time-dependent receiver operating characteristic (ROC) curve analysis was also performed by using ‘survival’, ‘surviminer’, ‘timeROC’ packages to assess the discrimination ability of the risk score. The risk score was further validated in the validation cohort and the whole sepsis patients in GSE65682 (test cohort). Furthermore, we compared the 28-day mortality rate of sepsis patients from the GSE95233 dataset who were classified as low- or high-risk based on risk score (GSE95233 cohort). We were unable to perform the Kaplan-Meier analysis because the GSE95233 dataset lacked “time to event” data. A ROC curve was depicted *via* ‘pROC’ package.

### Independent prognostic evaluation of the risk score and construction of nomogram

Clinical information (age and sex) was extracted from patients in the GSE65682 dataset. These variables and risk score were analyzed together in univariate and multivariate Cox regression analyses. The ‘rms’ package was used to create a nomogram based on independent variables for visualization and potential clinical use in predicting the prognosis of sepsis patients. The ROC curve and calibration curve were used to assess the nomogram’s performance.

### Functional enrichment analysis and immune infiltration analysis

We used ‘limma’ package again and based on the same criteria (log_2_|fold change| > 0.5 and adjust *P* value< 0.05) to screen DEGs between low- and high-risk groups in the sepsis patients from GSE65682. To explore the DEGs-related signal pathways and biological function, “clusterprofiler” package (25) was applied to perform the Kyoto Encyclopedia of Genes and Genomes (KEGG) and Gene Ontology (GO) enrichment analysis. To quantify the relative proportion of immune cell infiltration, we used the CIBERSORT algorithm (26) with 1000 permutations to calculate 22 types of immune cell composition for each sample. The composition of these immune cells between low- and high-risk groups was compared *via* wilcoxson test. In addition, we performed a correlation analysis between the differentially immune cells and the pyroptosis-related genes.

### Evaluation of the diagnostic value of the selected genes

We further investigated whether the selected prognostic pyroptosis-related genes also have potential value in the diagnosis of sepsis. The performance of these genes was evaluated using the ROC curves in the GSE65682 dataset and GSE95233 dataset.

### Statistical analysis

All statistical analyses were performed using R software (version 4.1.0) and R studio (Version 1.2.5042). The wilcoxon test was used to compare the gene expression level between sepsis patients and healthy individuals, and the composition of immune cells between low- and high-risk groups. Pearson chi-square test was applied to compare categorical variables. LASSO-Cox regression was used for candidate genes selection. The Kaplan-Meier method and log-rank test were used to compare the survival rate between the low- and high-risk groups. Univariate and multivariate cox regression analyses were used to assess the independent prognostic variables. A two-tailed *P* value<0.05 was considered statistically significant except for a certain *P* value was set.

## Results

### Identification of pyroptosis-related DEGs between sepsis patients and healthy individuals

**Figure 1:**
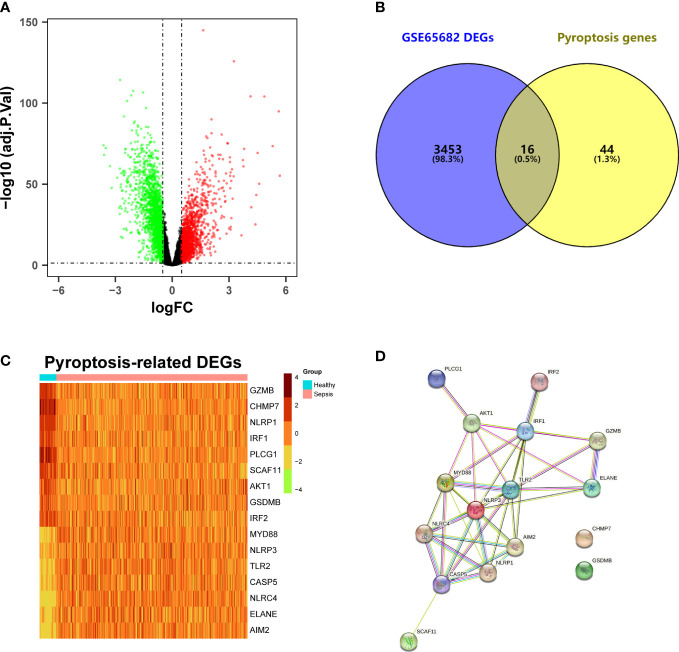
Identification of differentially expressed pyroptosis-related genes and interaction. **(A)** Volcano plot of DEGs in the GSE65682 dataset between sepsis patients and healthy individuals. **(B)** Venn plot of the DEGs and pyroptosis genes. **(C)** Heatmap of pyroptosis-related DEGs. **(D)** Protein-protein interaction (PPI) network analysis of proteins encoded by the pyroptosis-related DEGs.

A total of 3469 DEGs were identified from the GSE65682 dataset between sepsis and healthy samples, including 1571 upregulated genes and 1898 downregulated genes (**Figure 1A**; **Supplementary Table 2**). After intersecting with the pyroptosis-related genes, 16 pyroptosis-related DEGs were obtained (**Figure 1B**). Among them, the expression level of 7 genes (*MYD88, NLRP3, TLR2, CASP5, NLRC4, ELANE, AIM2*) were upregulated, while the expression level of 9 genes (*GZMB, CHMP7, NLRP1, IRF1, PLCG1, SCAF11, AKT1, GSDMB, IRF2*) were downregulated (**Figure 1C**). The result of the PPI analysis was presented in **Figure 1D**. There were 40 interaction relationships between these pyroptosis-related DEGs.

### Consensus clustering analysis based on the pyroptosis-related DEGs

**Figure 2:**
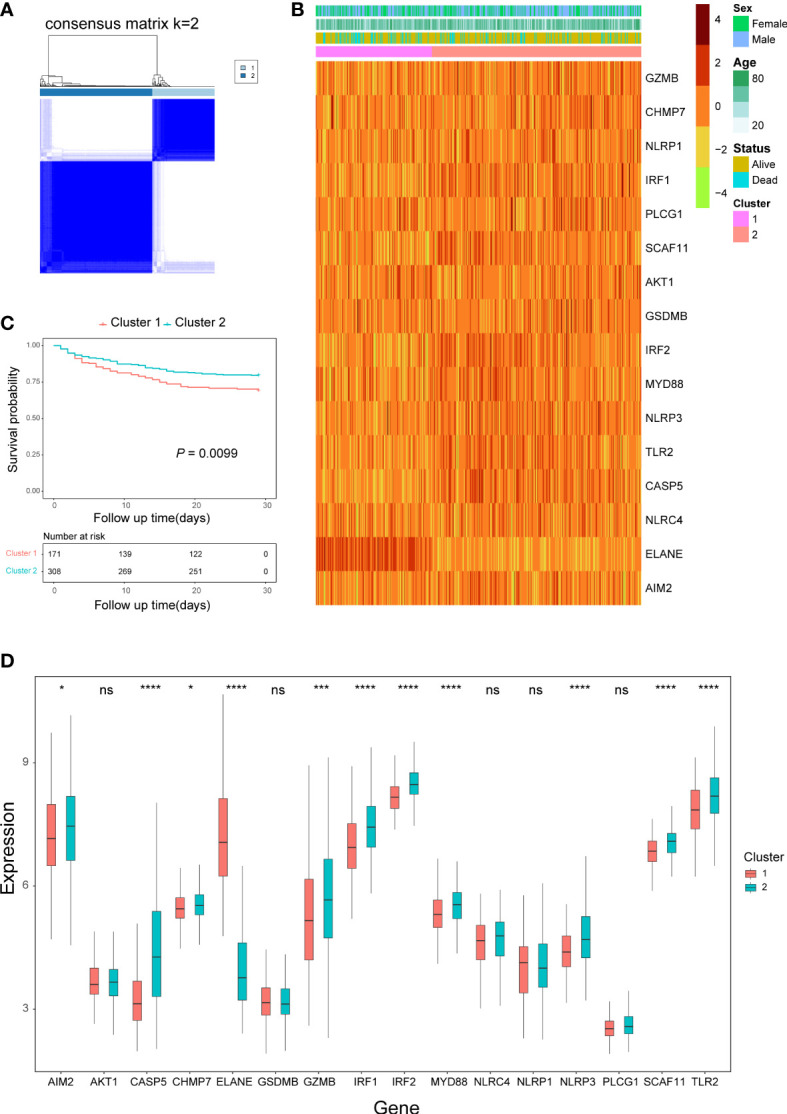
Consensus clustering analysis based on pyroptosis-related DEGs. **(A)** Consensus matrix when k=2. **(B)** Heatmap of pyroptosis-related DEGs expression and clinical characteristics in the two clusters. **(C)** Kaplan-Meier curves analysis for the survival of patients between the two clusters. **(D)** Box plots of pyroptosis-related DEGs expression level in the two clusters. ns, not significant; *P< 0.05,***P< 0.001, ****P< 0.0001.

According to the empirical CDF value, k = 2 was found to be the most acceptable point for the consensus cluster with the most distinct differences between clusters (**Figure 2A**; **Supplementary Figure 1**). Some of the expression levels of the pyroptosis-related DEGs were different between the two clusters (**Figures 2B, D**), and sepsis patients in cluster 1 had a worse prognosis than patients in cluster 2 (*P* = 0.0099) (**Figure 2C**).

### Development of a prognostic risk score based on pyroptosis-related DEGs

**Figure 3:**
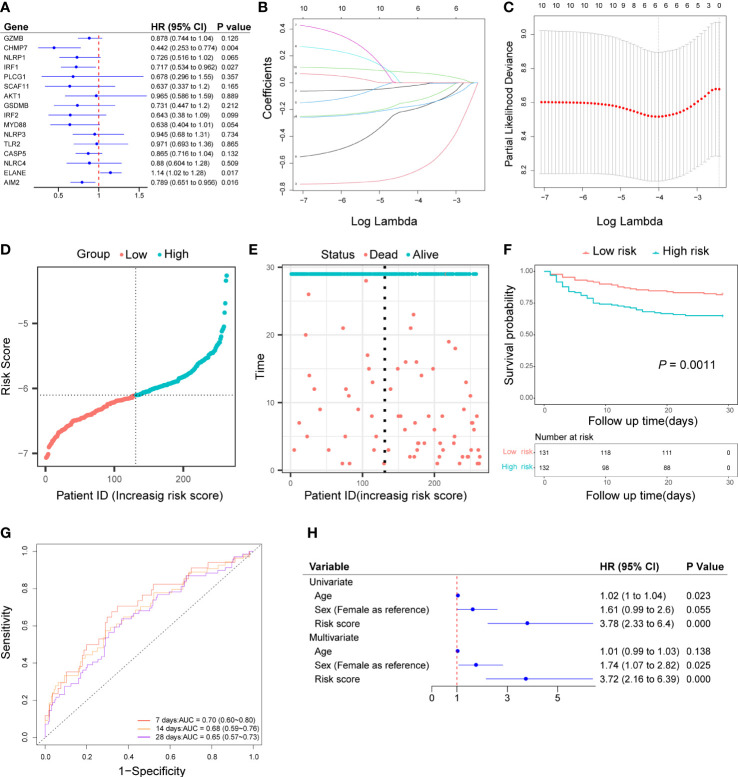
Construction of pyroptosis-related prognostic risk score and prediction of the prognosis in the discovery cohort. **(A)** Univariate cox regression analysis of survival for 16 pyroptosis-related DEGs, and 10 genes with a *P*< 0.2. **(B)** LASSO-Cox regression of the 10 candidate genes. **(C)** Cross-validation for tuning predictor selection. **(D)** Distribution of patients based on the risk score. **(E)** Survival time and status of patients. **(F)** Kaplan-Meier curves analysis for the survival of patients in low- and high-risk groups. **(G)** Time-dependent receiver operating characteristic curve for 7-, 14-, and 28-day survival of sepsis patients. **(H)** Univariate and multivariate cox regression.

Based on the univariate cox regression analysis, 10 out of 16 genes (*GZMB, CHMP7, NLRP1, IRF1, SCAF11, IRF2, MYD88, CASP5, ELANE, AIM2*) met *P<* 0.2 and were selected as prognostic candidate genes (**Figure 3A**). Of the 10 prognostic candidate genes, 1 gene (*ELANE*) was associated with increased risk with (HR > 1), while the remaining 9 genes (*GZMB, CHMP7, NLRP1, IRF1, SCAF11, IRF2, MYD88, CASP5*) were associated with lower risk (HR< 1). By performing LASSO-Cox regression analysis with the above candidate genes, a subset of six genes (*GZMB, CHMP7, NLPR1, MYD88, ELANE, AIM2*) were determined to develop a prognostic risk score based on the minimal criteria of λ (**Figures 3B, C**). The calculation of the risk score for each sample was according to the formula as follows: Risk score = [(-0.01470407 x *GZMB* expression value) + (-0.63970285 x *CHMP7* expression value) + (-0.12447921 x *NLRP1* expression value) + (-0.18172740 x *MYD88* expression value) + (0.07255413 x *ELANE* expression value) + (-0.16451424 x *AIM2* expression value)]. After calculating the median risk score, 263 patients were stratified into two risk groups (131 in the low-risk group and 132 in the high-risk group) (**Figure 3D**). Patients in the high-risk group had more death (**Figure 3E**) and the Kaplan-Meier curve showed that patients in the low-risk group had a higher survival rate than those in the high-risk group (*P* = 0.0011, **Figure 3F**). Time-dependent ROC analysis of the risk score revealed that the area under the curve (AUC) was 0.70 (95% CI: 0.60 to 0.80), 0.68 (95% CI: 0.59 to 0.76), and 0.65 (95% CI: 0.57 to 0.73) for 7-, 14-, and 28-day survival, respectively (**Figure 3G**). Both univariate and multivariate cox regression analysis indicated that the risk score was an independent predictor for prognosis in sepsis patients (Univariate: HR = 3.78, 95% CI: 2.33 to 6.4, *P*< 0.001; Multivariate: HR = 3.72, 95% CI: 2.16 to 6.39, *P*< 0.001; **Figure 3H**).

### Validation of the prognostic risk score

**Figure 4:**
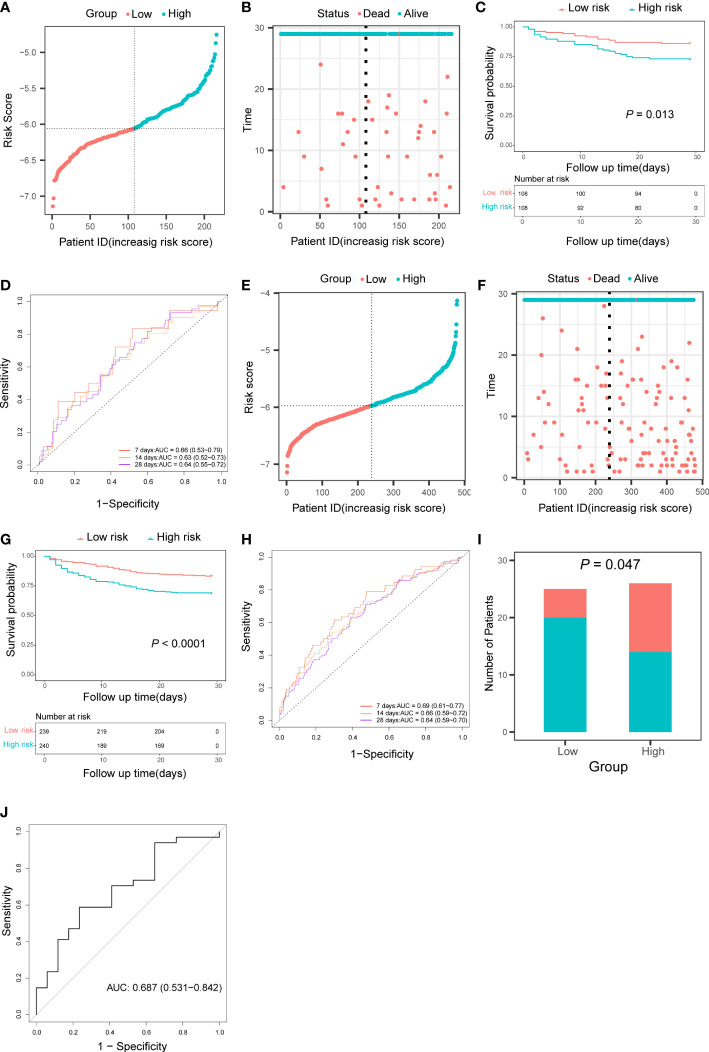
Validation of the pyroptosis-related prognostic risk score in the validation cohort, the test cohort, and the GSE95233 cohort. **(A, E)** Distribution of patients based on the risk score in the validation cohort **(A)** and the test cohort **(E)**. **(B, F)** Survival time and status of patients in the validation cohort **(B)** and the test cohort **(F)**. **(C, G)** Kaplan-Meier curves analysis for the survival of patients in low- and high-risk groups in the validation cohort **(C)** and the test cohort **(G)**. **(D, H)** Time-dependent receiver operating characteristic curve for 7-, 14-, and 28-day survival of patients in the validation cohort **(D)** and the test cohort **(H)**. **(I)** The survival of patients in low- and high-risk groups in the GSE95233 cohort. **(J)** Receiver operating characteristic curve for 28 days survival of patients in the GSE95233 dataset.

Patients in the validation cohort and the test cohort were divided into two groups based on the median risk score, respectively (**Figures 4A, E**). Patients in the high-risk groups also occurred more death events (**Figures 4B, F**) and Kaplan-Meier curve showed that patients in the low-risk group had significantly higher survival rates than those in the high-risk group (In the validation cohort: *P* = 0.013, **Figure 4C**; In the test cohort: *P<* 0.0001, **Figure 4G**). AUC of the time-dependent ROC curve was 0.66 (95% CI: 0.53 to 0.79), 0.63 (95% CI: 0.52 to 0.73), and 0.64 (95% CI: 0.55 to 0.72) for 7-, 14-, and 28-day survival in the validation cohort (**Figure 4D**) and 0.69 (95% CI: 0.61 to 0.77), 0.65 (95% CI: 0.59 to 0.72), and 0.64 (95% CI: 0.59 to 0.70) for 7-, 14-, and 28-day survival in the test cohort (**Figure 4H**). According to the result of univariate and multivariate cox regression, the risk score also could be an independent prognostic factor in sepsis patients in these two cohorts (**Supplementary Figure 2**). In addition, patients in the low-risk group also had a higher survival rate than those in the high-risk group in the GSE95233 cohort (*P* = 0.047, **Figure 4I**). The AUC under the ROC curve was 0.687 (95% CI: 0.531 to 0.842) (**Figure 4J**).

### Construction of a nomogram based on the risk score and independent clinical data

**Figure 5:**
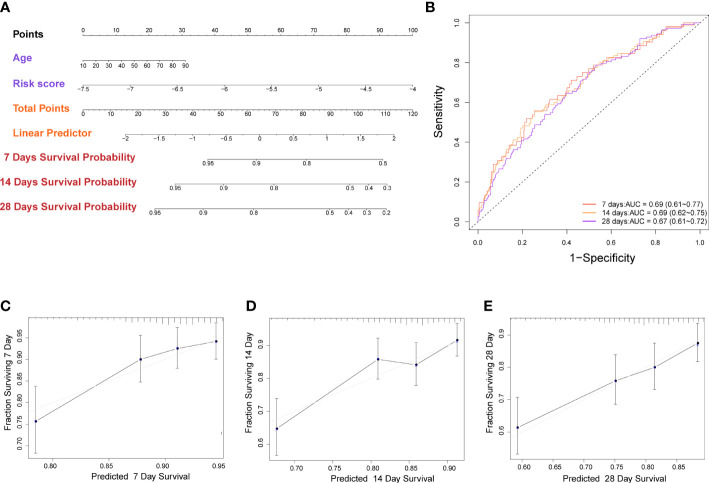
Construction of nomogram based on the pyroptosis-related prognostic risk score and clinical characteristic. **(A)** Nomogram for predicting 7-, 14-, and 28-day survival for sepsis patients. **(B)** Time-dependent receiver operating characteristic curves for 7-, 14-, and 28-day survival of patients. **(C-E)** Calibration curve of the nomogram for predicting 7-, 14-, and 28-day survival of patients.

In order to potentially clinically used the risk score and predicted more precisely the prognosis of sepsis patients. We created a nomogram using the patient’s age (*P* = 0.029, **Supplementary Figure 2B**) and risk score. (**Figure 5A**). The AUC of the nomogram for predicting 7-, 14-, and 28-day of survival were 0.69 (95% CI: 0.61 to 0.77), 0.69 (95% CI: 0.62 to 0.75), and 0.67 (95% CI: 0.61 to 0.72) (**Figure 5B**). The calibration curve indicated a good calibration between predicting probability and actual probability (**Figures 5C–E**).

### Functional enrichment analysis between different risk groups

**Figure 6:**
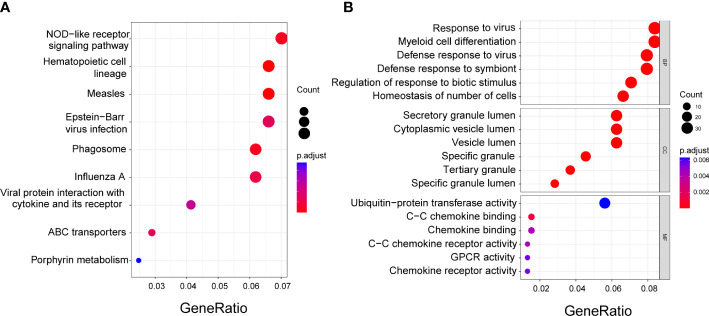
Functional enrichment analysis of the DEGs between low- and high-risk groups. **(A)** Kyoto Encyclopedia of Genes and Genomes (KEGG) analysis. **(B)** Gene Ontology (GO) enrichment analysis. BP, Biological process; CC, Cellular component; MF, Molecular function.

A total of 485 DEGs were identified between the low- and high-risk groups. Among them, 164 genes were downregulated and 321 genes were upregulated (**Supplementary Table 3**). On the basis of these DEGs, KEGG and GO analyses were performed. The results showed that the DEGs were mainly correlated with NOD-like receptor signaling pathway, hematopoietic cell lineage, and response to the virus (**Figures 6A, B**).

### Comparison of immune infiltration between different risk groups

**Figure 7:**
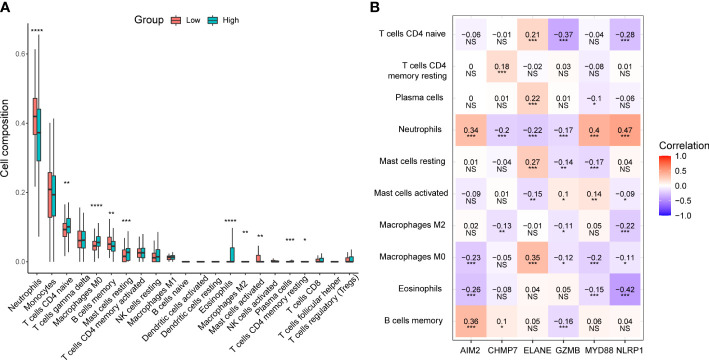
Immune infiltration analysis between low- and high-risk groups in the GSE65682 dataset. **(A)** Box plots of 22 types of immune cell composition between low- and high-risk groups. *P< 0.05; **P< 0.01; ***P< 0.001; ****P< 0.0001. **(B)** The correlation between the selected six pyroptosis-related genes and the immune cells. NS, Not significant, *P< 0.05; **P< 0.01; ***P< 0.001.

Based on the results of the functional analysis. We further compared immune infiltration between low- and high-risk groups by using the CIBERSORT algorithm. In the low-risk group, neutrophils, B cells memory, and mast cells activated were significantly higher than that in the high-risk group, while T cells CD4 naive, macrophages M0, mast cells resting, eosinophils, macrophages M2, plasma cells, and T cells CD4 memory resting were significantly lower (**Figure 7A**). The correlation analysis revealed that pyroptosis-related genes were significantly associated with many immune cells. All six genes were correlated with neutrophils, with *AIM2* (r = 0.34), *MYD88* (r = 0.4), and *NLRP1* (r = 0.47) showing a positive correlation, and *CHMP7* (r = -0.2), *ELANE* (r = -0.22), and *GZMB* (r = -0.17) showing a negative correlation (**Figure 7B**).

### Performance of the selected pyroptosis-related genes in the diagnosis of sepsis

**Figure 8:**
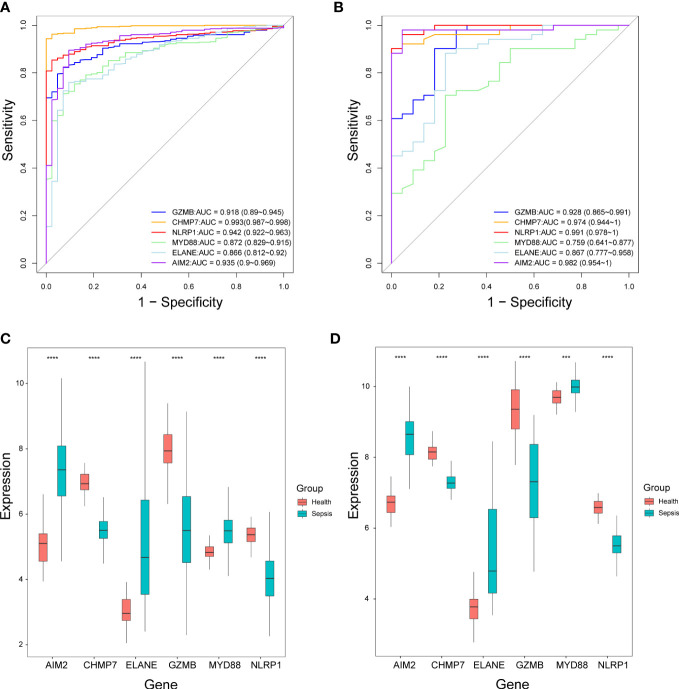
The performance of the selected six pyroptosis-related genes in the diagnosis of sepsis. **(A, B)** Receiver operating characteristic curves in the GSE65682 dataset **(A)** and GSE95233 dataset **(B)**. AUC, Area under the curve. **(C, D)** Box plots of the expression levels of the six genes between sepsis patients and healthy individuals in the GSE65682 dataset **(C)** and GSE95233 dataset **(D)**. ***P< 0.001, ****P< 0.0001.

According to the ROC curves, four out of six genes (*GZMB, CHMP7, NLRP1*, and *AIM2*) had good diagnostic value in the diagnosis of sepsis, with AUC > 0.9 in both the GSE65682 and GSE95233 datasets (**Figures 8A, B**). In both datasets, the expression level of *AIM2*, *ELANE*, and *MYD88* were significantly higher in sepsis patients compared to healthy individuals, while the expression level of *CHMP7*, *GZMB*, and *NLRP1* were significantly lower. (**Figures 8C, D**).

## Discussion

Over the past two decades, many biomarkers have been identified for sepsis including inflammatory factors, cell proteins, and miRNA (27). However, early diagnosis and prediction of the prognosis of sepsis are still difficult due to the complicated etiology, ambiguous pathogenic microorganisms, and early non-specific clinical signs of sepsis patients. Hence, it is still important to explore new biomarkers and provide new insight. In this study, we first identified 16 pyroptosis-related DEGs between sepsis patients and healthy individuals. Two pyroptosis-related sepsis clusters were identified and patients in cluster 1 had a poor prognosis than patients in cluster 2. To further explore the prognostic value of these genes, we constructed a prognostic risk score based on six genes’ signatures *via* univariate cox regression analysis and LASSO-Cox regression analysis and validated its performance in external cohorts. After that, a nomogram was constructed based on the risk score and clinical information for clinical use. Functional enrichment analysis revealed that the DEGs between the low- and high-risk groups were related to immune-related pathways, and immune infiltration analysis revealed a significant difference in immune status between the two groups. Finally, we found that four out of the six genes also have diagnostic value for sepsis.

Pyroptosis plays a dual role in anti-infection and pro-inflammatory in sepsis. On the one hand, pyroptosis damaged the intracellular pathogen’s living environment, reducing pathogen reproduction, and allowing intracellular pathogens to be removed and cleared by immune cells (14). On the other hand, the inflammatory factors such as IL-18 and IL-1β released by pyroptosis and the damaged tissue may contribute to cytokine storm cascade. Moderate pyroptosis may play a protective role against pathogens, while excessive pyroptosis may cause uncontrolled cytokine storms (20). Therefore, pyroptosis may be significantly associated with the prognosis of sepsis. As a result of the present study, we found that pyroptosis-related genes could be used to cluster patients with sepsis, and patients in the different clusters had different prognoses, suggesting that pyroptosis in patients with sepsis may be different, which may lead to a different prognosis. Then, we developed a prognostic risk score with six pyroptosis-related genes, including *GZMB, CHMP7, NLRP1, MYD88, ELANE*, and *AIM2*, and found that it could predict the prognosis of sepsis patients.

Granzymes B (*GZMB*) is a member of grazymes family that was considered to exert cytotoxic effects against pathogen invasion (28). Recent studies reported that *GZMB* is involved in the coagulation cascade, regulating the function of platelets and endothelial barrier permeability in sepsis (29). The expression level of GZMB in sepsis may be associated with the underlying pathogen. An upregulation of *GZMB* was found in patients with gram-negative bacterial infection (30), while a downregulation of *GZMB* was found in sepsis patients caused by burns and trauma (31). Charged multivesicular body protein 7 (*CHMP7*) is a part of the endosomal sorting complex required for transport III (ESCRT-III), which take part in the process of nuclear envelope formation, endosomal sorting, neurodevelopment, and attention deficit hyperactivity disorder (ADHD) (32). Our results revealed that the expression of *CHMP7* was significantly lower in sepsis patients and a higher expression of *CHMP7* was associated with a better prognosis. Due to the limited number of studies, the role of *CHMP7* in sepsis remained unclear and our study may provide some insights for future study. The NLR family pyrin domain containing 1(*NLRP1*) and absent in melanoma 2 (*AIM2*) are pathogen pattern recognition (PRR) in the intracellular that respond to the pathogen-associated molecular patterns (PAMPs) or danger-associated molecular patterns (DAMPs), and activation caspase-1 mediated canonical pyroptosis pathway. Previous publications have found a significantly lower expression of *NLRP1* and a significantly higher expression of *AIM2* among sepsis patients (33, 34), besides, an even lower expression of *NLRP1* was found in the non-survivor (33). Our results were consistent with these findings and we also showed that a higher expression of *AIM2* is associated with a better prognosis. Further studies are still needed to clarify why the expression patterns of those genes are different even though they trigger the same canonical pathway of pyroptosis. Another PRR that is located in the cellular membrane called Toll-like receptor (TLR) is also involved in the recognition of PAMPs. Except for TLRP3, most TLR started its inflammatory response *via* a common signaling pathway by recruitment signaling adaptor protein including myeloid differentiation primary response protein (*MYD88*) (35). An overexpression of *MYD88* was associated with a poor prognosis of neonatal sepsis (36); However, our study found that a higher expression of *MYD88* was associated with a better prognosis. This discrepancy may be attributed to the difference in the immune system between adults and neonatal. *ELANE* encodes neutrophil elastase that is secreted by neutrophil. Neutrophil elastase could cleave GSDMD and cause neutrophil death, and the level of *ELANE* was reported to be associated with the severity of sepsis (37, 38). The expression level of *ELANE* was significantly higher in sepsis patients and associated with poor prognosis in our study.

Sepsis-induced both innate and adaptive immune dysfunction. The immune status of sepsis patients may be a crucial factor affecting the prognosis of sepsis (39, 40). The functional enrichment and immune infiltration analyses revealed differences in immune-related pathways and immune cell composition between the patients in the low-and high-risk groups, which may explain why the two risk groups have different prognoses. Besides, these findings also suggested that pyroptosis plays a role in immune dysfunction. The immune cell could also occur pyroptosis, which has been considered to play an essential role in the progression of sepsis. The regulation of immune cell pyroptosis has been shown to improve the prognosis of sepsis, with many studies focused on the regulation of macrophage pyroptosis (41). For example, Luo et al. found that inhibiting macrophage pyroptosis by Platelet endothelial cell adhesion molecule-1 (PECAM-1) could improve the prognosis in a septic murine model (42) and Song et al. reported Sphingosine-1-phosphate receptor 2 (S1PR2) knockout could reduce macrophage pyroptosis and improve sepsis outcome in mice (43). Notably, the correlation analysis showed that all six pyroptosis-related genes were correlated with neutrophils, implying that pyroptosis and neutrophils are closely related. Neutrophils constitute the majority of immune cells in human peripheral blood and play an important role in pathogen recognition and clearance. The role of neutrophil pyroptosis in sepsis remains unclear, and regulation of neutrophil pyroptosis has recently been assumed to have potential therapeutic value in sepsis (44, 45). Therefore, we believe that neutrophil pyroptosis in sepsis could be considered for further investigation in future studies.

There are several limitations that should be acknowledged in our study. First, although the prognostic risk score showed good performance, it still needs to be validated in large prospective cohort studies. Second, the prognostic factors from the GEO database were insufficient since other prognostic factors such as comorbidity diseases and infectious organisms were not included. Third, the molecular mechanism of the pyroptosis-related genes interacting with the immune cells needs to be further explored in the experimental study.

In summary, we developed and validated a novel prognostic predictive risk score for sepsis based on six pyroptosis-related genes. The risk score was an independent prognostic factor of sepsis prognosis. Four out of the six genes also have potential diagnostic value in sepsis diagnosis. Our findings may provide new insight into the role of pyroptosis in sepsis and serve as a foundation for future research.

## Data availability statement

The datasets presented in this study can be found in online repositories. The names of the repository/repositories and accession number(s) can be found in the article/**Supplementary Material**.

## Author contributions

SL and WZ designed the study. SL, MX, XC, JP and ZS collected, analyzed, and interpreted the data. SL wrote the manuscript, WZ reviewed and revised the manuscript. All authors contributed to this study and approved the submitted version of the manuscript.

## Supplementary material

Supplementary Figure 1 Consensus clustering analysis based on pyroptosis-related DEGs. **(A)** Consensus empirical CDF. **(B)** Delta area.

Supplementary Figure 2 Univariate and multivariate cox regression of the prognostic predictors. **(A)** In the validation cohort. **(B)** In the test cohort.
