# Supplementary figures and images for: Predicting the prognosis in patients with sepsis by a pyroptosis-related gene signature
Source: Front Immunol. 2022 Dec 21;13:1110602. doi: 10.3389/fimmu.2022.1110602 (PMC9811195; doi:10.3389/fimmu.2022.1110602)

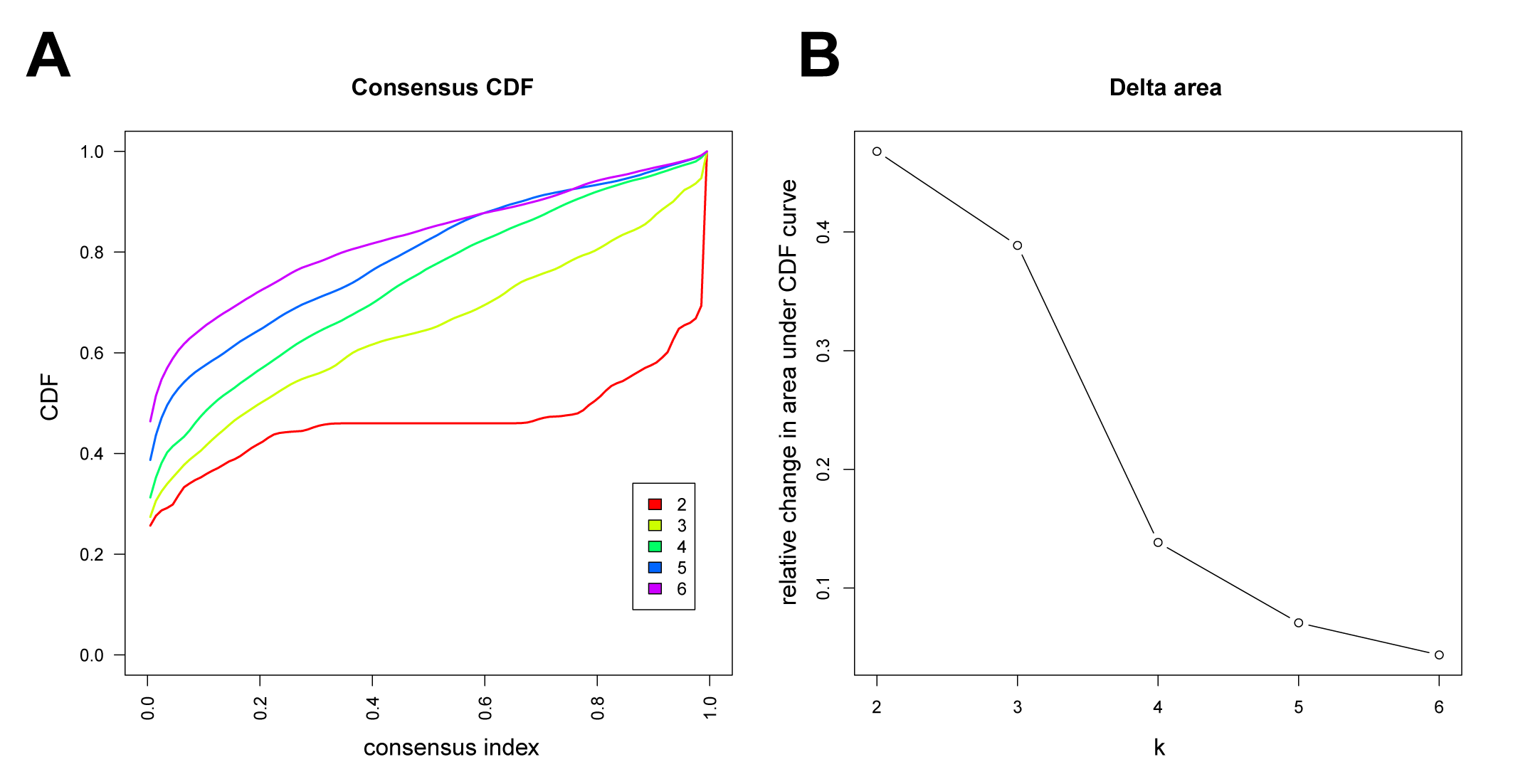

Supplement: Supplementary Figure 1 — Consensus clustering analysis based on pyroptosis-related DEGs. (A) Consensus empirical CDF. (B) Delta area. [file Image_1.tif]

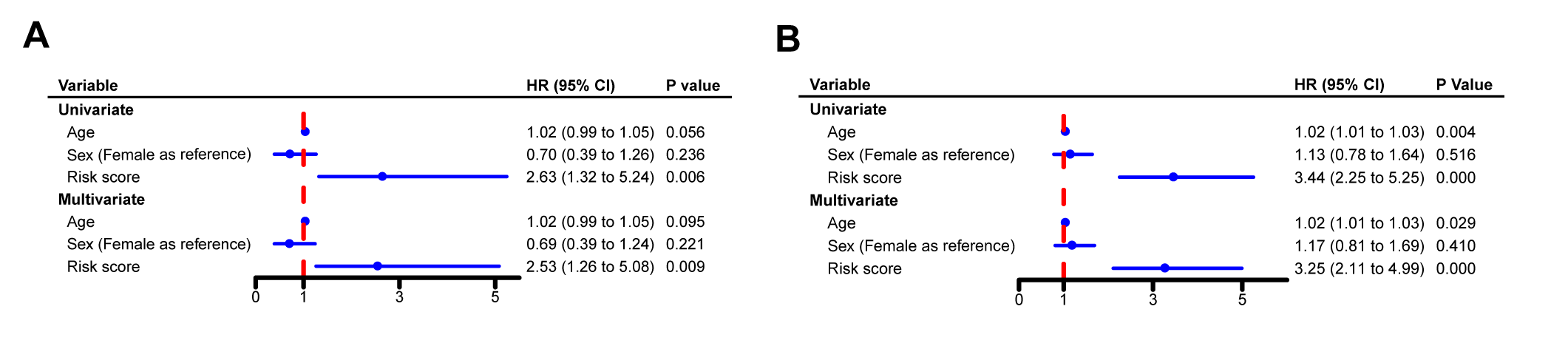

Supplement: Supplementary Figure 2 — Univariate and multivariate cox regression of the prognostic predictors. (A) In the validation cohort. (B) In the test cohort. [file Image_2.tif]
