# Supplementary material for: Predicting the prognosis in patients with sepsis by a pyroptosis-related gene signature
Source: Front Immunol. 2022 Dec 21;13:1110602. doi: 10.3389/fimmu.2022.1110602 (PMC9811195; doi:10.3389/fimmu.2022.1110602)
Supplement: Supplementary file 3 [file Table_1.docx]

**Supplementary Table 1 Pyroptosis-related genes**

| **Number** | **Gene symbol** | **Full name** |
| --- | --- | --- |
| 1 | AIM2 | Absent in melanoma 2 |
| 2 | AKT1 | AKT Serine/Threonine Kinase 1 |
| 3 | BAK1 | BCL2 Antagonist/Killer 1 |
| 4 | BAX | BCL2 Associated X |
| 5 | CASP1 | Cysteine-aspartic acid protease-1 |
| 6 | CASP3 | Cysteine-aspartic acid protease-3 |
| 7 | CASP4 | Cysteine-aspartic acid protease-4 |
| 8 | CASP5 | Cysteine-aspartic acid protease-5 |
| 9 | CASP6 | Cysteine-aspartic acid protease-6 |
| 10 | CASP8 | Cysteine-aspartic acid protease-8 |
| 11 | CASP9 | Cysteine-aspartic acid protease-9 |
| 12 | CHMP2A | Charged multivesicular body protein 2A |
| 13 | CHMP2B | Charged multivesicular body protein 2B |
| 14 | CHMP3 | Charged multivesicular body protein 3 |
| 15 | CHMP4A | Charged multivesicular body protein 4A |
| 16 | CHMP4B | Charged multivesicular body protein 4B |
| 17 | CHMP4C | Charged multivesicular body protein 4C |
| 18 | CHMP6 | Charged multivesicular body protein 6 |
| 19 | CHMP7 | Charged multivesicular body protein 7 |
| 20 | CXCL8 | C-X-C Motif Chemokine Ligand 8 |
| 21 | CYCS | Cytochrome c, somatic |
| 22 | ELANE | Elastase, neutrophil expressed |
| 23 | GPX4 | Glutathione peroxidase 4 |
| 24 | GSDMA | Gasdermin A |
| 25 | GSDMB | Gasdermin B |
| 26 | GSDMC | Gasdermin C |
| 27 | GSDMD | Gasdermin D |
| 28 | GSDME | Gasdermin E |
| 29 | GZMB | Granzyme B |
| 30 | HMGB1 | High mobility group box 1 |
| 31 | IL18 | Interleukin 18 |
| 32 | IL1A | Interleukin 1, alpha |
| 33 | IL1B | Interleukin 1 beta |
| 34 | IL6 | Interleukin 6 |
| 35 | IRF1 | Interferon regulatory factor 1 |
| 36 | IRF2 | Interferon regulatory factor 2 |
| 37 | JUN | Jun Proto-Oncogene, AP-1 Transcription Factor Subunit |
| 38 | MYD88 | Myeloid differentiation primary response protein |
| 39 | NFKB1 | Nuclear Factor Kappa B Subunit 1 |
| 40 | NLRC4 | NLR family CARD domain containing 4 |
| 41 | NLRP1 | NLR family pyrin domain containing 1 |
| 42 | NLRP2 | NLR family pyrin domain containing 2 |
| 43 | NLRP3 | NLR family pyrin domain containing 3 |
| 44 | NLRP6 | NLR family pyrin domain containing 6 |
| 45 | NLRP7 | NLR family pyrin domain containing 7 |
| 46 | NOD1 | Nucleotide binding oligomerization domain containing 1 |
| 47 | NOD2 | Nucleotide binding oligomerization domain containing 2 |
| 48 | PJVK | Pejvakin/deafness, autosomal recessive 59 |
| 49 | PLCG1 | Phospholipase C gamma 1 |
| 50 | PRKACA | Protein kinase cAMP-activated catalytic subunit alpha |
| 51 | PTGS2 | Prostaglandin-Endoperoxide Synthase 2 |
| 52 | PYCARD | PYD and CARD domain containing |
| 53 | SCAF11 | SR-related CTD associated factor 11 |
| 54 | STAT3 | Signal Transducer And Activator Of Transcription 3 |
| 55 | TIRAP | TIR domain containing adaptor protein |
| 56 | TLR2 | Toll Like Receptor 2 |
| 57 | TLR9 | Toll Like Receptor 9 |
| 58 | TNF | Tumor necrosis factor |
| 59 | TP53 | Tumor protein p53 |
| 60 | TP63 | Tumor protein p63 |
